# The nutritional functions of dietary sphingomyelin and its applications in food

**DOI:** 10.3389/fnut.2022.1002574

**Published:** 2022-10-19

**Authors:** Fang Yang, Guoxun Chen

**Affiliations:** ^1^School of Laboratory Medicine, Hubei University of Chinese Medicine, Wuhan, China; ^2^Department of Nutrition, The University of Tennessee, Knoxville, TN, United States

**Keywords:** sphingolipids, sphingomyelin, structure, metabolism, digestion, chronic metabolic diseases, applications

## Abstract

Sphingolipids are common structural components of cell membranes and are crucial for cell functions in physiological and pathophysiological conditions. Sphingomyelin and its metabolites, such as sphingoid bases, ceramide, ceramide-1-phosphate, and sphingosine-1-phosphate, play signaling roles in the regulation of human health. The diverse structures of sphingolipids elicit various functions in cellular membranes and signal transduction, which may affect cell growth, differentiation, apoptosis, and maintain biological activities. As nutrients, dietary sphingomyelin and its metabolites have wide applications in the food and pharmaceutical industry. In this review, we summarized the distribution, classifications, structures, digestion, absorption and metabolic pathways of sphingolipids, and discussed the nutritional functioning of sphingomyelin in chronic metabolic diseases. The possible implications of dietary sphingomyelin in the modern food preparations including dairy products and infant formula, skin improvement, delivery system and oil organogels are also evaluated. The production of endogenous sphingomyelin is linked to pathological changes in obesity, diabetes, and atherosclerosis. However, dietary supplementations of sphingomyelin and its metabolites have been shown to maintain cholesterol homeostasis and lipid metabolism, and to prevent or treat these diseases. This seemly paradoxical phenomenon shows that dietary sphingomyelin and its metabolites are candidates for food additives and functional food development for the prevention and treatment of chronic metabolic diseases in humans.

## Introduction

Phospholipids act as the main component of biological membranes, emulsifier and surfactant. There are two types of phospholipids. The first one is glycerophospholipids with a glycerol backbone such as phosphatidylcholine, phosphatidylethanolamine, phosphatidylserine, phosphatidylglycerol, phosphatidylinositol, cardiolipin, plasmalogen, phosphatidic acid and platelet activating factor (1). The other one is sphingolipids with sphingosine, the second largest group of membrane lipids. Sphingolipids such as ceramides, sphingomyelin, and glycosphingolipids have a polar head group and two non-polar tails (a long hydrocarbon fatty acyl group and a sphingoid base backbone) (2). Glycerophospholipids are important amphiphilic substances due to the presence of a hydrophilic head composed of substituent groups linked by phosphoric acid and a hydrophobic tail composed of fatty acyl groups. Currently, the LIPID MAPS database includes more than 4900 known sphingolipids, which are commonly found in all viruses, prokaryotes and eukaryotes (3). Sphingolipids are mainly found in membranes of cells, where the hydrophobic tail of phospholipids and other membrane lipids such as cholesterol are embedded. Both plasma and organelle membranes contain sphingomyelin, which distributes symmetrically throughout the membrane’s bilayer. Contrarily, sphingolipids that are glycosylated (glycosphingolipids) are often arranged asymmetrically with their saccharide moieties facing the extracellular environment (4). Since the discovery in the 1880s (5), sphingolipids have been located on cell membranes, lipoproteins and other lipid-rich tissue structures, and are considered very important for maintaining membrane integrity, lipid raft formation, cell metabolism and signal transduction, cell growth, differentiation and apoptosis (6, 7). In foods, sphingolipids are present in dairy products, egg, meat product and soybeans, and some fruits and vegetables in small amounts (8).

The *de novo* synthesis of sphingolipids begins with the formation of ceramides, which is derived from serine and palmitate in a process that consists of condensation, reduction, acylation, and desaturation in the endoplasmic reticulum (ER). Then, ceramides are transported to the Golgi apparatus for the formations of sphingomyelin, ceramide-1-phosphate (C1P), inositol phosphorylceramides and glycosphingolipids (9–11). Sphingomyelin is one of the most important sphingolipids in animal tissues. Sphingomyelin from sources varies in sphingosine [long-chain bases (LCBs)] and fatty acyl groups (12). Sphingomyelin co-exists with phosphatidylcholine on the outer leaflet of the cell membrane. The dietary sphingomyelin is digested by intestinal alkaline SMase (Alk-SMase) and neutral ceramidase (N-CDase), and eventually hydrolyzed to ceramides, phosphorylcholine, sphingosine and fatty acids in the small intestine (3, 13). Unlike sphingomyelin and ceramides, sphingosine can be absorbed intact into intestinal mucosal cells and converted to sphingosine-1-phosphate (S1P), ceramides, sphingomyelin and glycosphingolipids, which are transported with chylomicrons into the lymph circulation and then the blood circulation, and newborn high-density lipoproteins (HDL) into the blood circulation (3, 13–15). The sphingomyelin metabolism and its plasma level are also affected by the types of fatty acids in the diet, vitamin B6, vitamin C, vitamin D, and vitamin K (16–23). The endogenous sphingomyelin is involved in adipose tissue function, obesity, diabetes and atherosclerosis related pathology (24–27), while exogenous dietary sphingomyelin may be beneficial for regulating cholesterol homeostasis and lipid metabolism, and for the prevention and treatment of chronic metabolic diseases (28–32). Due to the nutritional functions of dietary sphingomyelin and its metabolites, it has broad application prospects in food industry.

## Classification and structures of sphingolipids

### Classification of sphingolipids

When Johann Ludwig Wilhelm Thudichum isolated sphingolipids from the nerve tissues in 1880s, he named the molecule “sphingosine” after the Greek mythological creature sphinx, the beast of ancient Egyptian mythology that loved to present many enigmas to the inquirers (5). The LIPID MAPS Lipid Classification System classifies lipids into eight categories, fatty acyls, glycerolipids, glycerophospholipids, sphingolipids, sterol lipids, prenol lipids, saccharolipids, and polyketides. As shown in Table 1, sphingolipids have the most structural diversity and are subdivided into sphingoid bases, ceramides, phosphosphingolipids, phosphonosphingolipids, neutral glycosphingolipids, acidic glycosphingolipids, basic glycosphingolipids, amphoteric glycosphingolipids, arspidenosphingolipids and other sphingolipids (2). Sphingoid bases that include sphingosines, sphinganines, phytosphingosines, sphingoid base homologs and variants (C14, C16, and C17), sphingoid base S1P, lysosphingomyelins and lyso(glyco)sphingolipids, *N*-methylated sphingoid bases and sphingoid base analogs are the backbone of sphingolipids. A ceramide is generated when a fatty acyl group is linked to the sphingoid bases through an amide-linkage. There are ceramides, dihydroceramides, phytoceramides, acylceramides and its phosphate derivative C1P. Phosphosphingolipids such as sphingomyelins, ceramide phosphoethanolamines and ceramide phosphoinositols, are formed when the head group such as phosphocholine is linked to the ceramide via a phosphodiester linkage. In addition, simple and complex glycosphingolipids can be formed when mono or polysaccharides are attached to ceramide via glycosidic bonds such as in cerebrosides and gangliosides, respectively. Based on the polar parts, glycosphingolipids can be classified into three categories neutral, acidic, alkaline and amphoteric glycosphingolipids.

**TABLE 1:** Classification and representative structures for sphingolipids.

Common name	Systematic name	Formula	Structure
**Sphingoid bases**			
Sphingosine	Sphing-4-enine	C_18_H_37_NO_2_	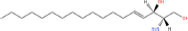
Sphinganine	Sphinganine	C_18_H_39_NO_2_	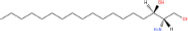
Phytosphingosines	4R-hydroxysphinganine	C_18_H_39_NO_3_	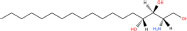
C16 Sphinganine	Hexadecasphinganine	C_16_H_35_NO_2_	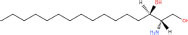
Sphingosine-1-phosphate	Sphing-4-enine-1-phosphate	C_18_H_38_NO_5_P	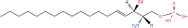
Sphingosine-1-phosphocholine	Sphing-4-enine-1-phosphocholine	C_23_H_49_N_2_O_5_P	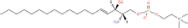
*N,N*-dimethylsphingosine	*N,N*-dimethylsphing-4-enine	C_20_H_41_NO_2_	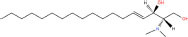
**Ceramide**			
Ceramide	*N*-acyl-sphing-4-enine	–	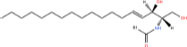
Dihydroceramide	*N*-acyl-sphinganine	–	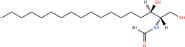
Cer(t18:0/16:0)	*N*-(hexadecanoyl)-4R-hydroxysphinganine	C_34_H_69_NO_4_	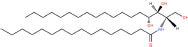
Omega-linoleoyloxy-Cer(d18:1/30:0)	*N*-(30-(9Z,12Z-octadecadienoyloxy)-triacontanoyl)-sphing-4-enine	C_66_H_125_NO_5_	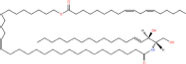
Ceramide-1-phosphate	*N*-(acyl)-sphing-4-enine-1-phosphate	–	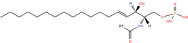
**Phosphosphingolipids**			
Sphingomyelin	*N*-acyl-sphing-4-enine-1-phosphocholine	–	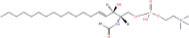
*N*-Acyl ceramide phosphoethanolamine	*N*-(acyl)-sphing-4-enine-1-phosphoethanolamine	–	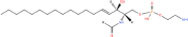
PI-Cer(d18:1/22:0)	*N*-(docosanoyl)-sphing-4-enine-1-phospho-(1′-myo-inositol)	C_46_H_90_NO_11_P	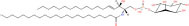
**Phosphonosphingolipids**			
Ceramide ciliatine	*N*-(acyl)-sphing-4-enine-1-(2-aminoethylphosphonate)	–	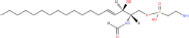
**Neutral glycosphingolipids**			
Glucosyl sphingosine	1-β-glucosyl-sphing-4-enine	C_24_H_47_NO_7_	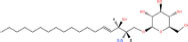
Gb3(d18:1/16:0)	Galα1-4Galβ1-4Glcβ-Cer(d18:1/16:0)	C_52_H_97_NO_18_	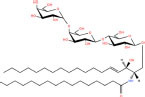
Asialo-GM2(d18:1/16:0)	GalNAcβ1-4Galβ1-4Glcβ-Cer(d18:1/16:0)	C_54_H_100_N_2_O_18_	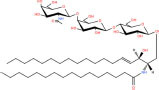
Lc3Cer(d18:1/16:0)	GlcNAcβ1-3Galβ1-4Glcβ-Cer(d18:1/16:0)	C_54_H_100_N_2_O_18_	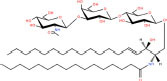
**Acidic glycosphingolipids**			
Gangliosides GM4(d18:1/16:0)	NeuAcα2-3Galβ-Cer(d18:1/16:0)	C_51_H_94_N_2_O_16_	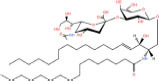
Sulfoglycosphingolipids Galbeta-Cer(d18:1/18:0)	*N*-octadecanoyl-1-β-(3′-sulfo)-glucosyl-sphing-4-enine	C_42_H_81_NO_11_S	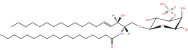
Glucuronosphingolipids GlcAbeta-Cer(d18:1/18:0)	*N*-(octadecanoyl)-1-β-glucuronosyl-sphing-4-enine	C_42_H_79_NO_9_	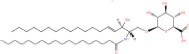
**Basic glycosphingolipids**			
Psychosine	1-β-galactosyl-sphing-4-enine	C_24_H_47_NO_7_	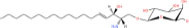
**Amphoteric glycosphingolipids**			
Psychosine sulfate	(2S,3R,4E)-2-amino-3-hydroxyoctadec-4-en-1-yl β-D-galactopyranoside 6-(hydrogen sulfate)	C_24_H_47_NO_10_S	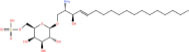

### Structures of sphingolipids

Studies have identified more than 600 lipid species from 23 lipids classes and more than 2,300 novel features (33). The sphingoid base backbone is synthesized *de novo* from serine and a fatty acid. After a fatty acyl chain is added to the amine group of sphingoid bases, sphingosines, sphinganine and phytosphingosine are transformed to ceramide, dihydroceramide, and phytoceramide, respectively. The attachment of hydrophilic head groups to the OH-group of C-1 of the ceramide yields phosphosphingolipids, phosphonosphingolipids, glycosphingolipids, and other species, including protein adducts (34). Naturally derived sphingoid bases vary in alkyl chain length and branching, the level of saturation, the number of hydroxyl groups and other characteristics (35–37). The variations of sphingosine bases, fatty acyl and hydrophilic head groups together make them the most diverse and complex lipid class. As shown in Table 1, sphingosine has a double bond at the C-4 position, and sphinganine and phytosphingosine are fully saturated, while phytosphingosine has an extra hydroxyl group. The abbreviations “d” and “t” seen in the shorthand nomenclature in Table 1 is to indicate the number of hydroxyl groups followed by the number of carbon atoms in the backbone and the number of double bonds in the fatty acyl group, while “d” is for the two (di-) hydroxyls group and “t” (tri-) for the additional hydroxyl group (38). So, sphingosine, sphinganine and phytosphingosine are designated as d18:1, d18:0, and t18:0, respectively. In addition, sphingoid base also includes the 1-phosphates, lysosphingolipids and *N*-methyl derivatives. The fatty acyl groups of ceramides are typically saturated or monounsaturated with various chain length from 14 to 36 carbons, while ceramides with C16, C18, and C24 are the most common ones in mammalian cells (2, 39, 40). When head groups (phosphate, phosphocholine or carbohydrate) are attached to ceramides at the C1-hydroxyl position, more complex sphingolipids such as C1P, phosphosphingolipids or glycosphingolipids are created (39). *N*-acylation of sphingosine creates dihydroceramides with C14 to C26 or in rare cases even up to C36 fatty acyl groups. The saturated or monounsaturated fatty acyl groups can be modified with an α and one additional hydroxylation. Sphingomyelin is composed of a sphingosine, a fatty acyl group and a phosphorylcholine head group (41). Its overall structure is roughly equivalent to replacing glycerol and a fatty acyl group in glycerophospholipid with sphingosine.

Glycosphingolipids can be divided into four sub-classes according to the types of carbohydrates and additional substituents, including neutral glycosphingolipids, acidic glycosphingolipids, basic glycosphingolipids and amphoteric glycosphingolipids (2). Neutral glycosphingolipids, also known as cerebrosides, contain one or more uncharged sugars, such as glucose (Glc), galactose (Gal) or N-acetylgalactosamine (GalNAc), *N*-acetylglucosamine (GlcNAc) and fucose. Acidic glycosphingolipids contain ionized groups adjoined to neutral or charged carbohydrates such as sialic acid to form sulfoglycosphingolipids, glucuronosphingolipids and gangliosides. Gangliosides contain negatively charged sialic acids (N-acetylneuraminic acid or N-glycolylneuraminic acid), and are named and classified according to the number of sialic acid residues attached (M/D/T representing gangliosides containing 1, 2, or 3 sialic acids, respectively) and inner sugar moieties such as Glc, Gal or GalNAc connected to ceramides. For example, the subscripts 1, 2, 3, 4 of gangliosides usually can be expressed as: 1 is Gal-GalNAc-Gal-Glc-ceramide, 2 is GalNAc-Gal-Glc-ceramide, 3 is Gal-Glc-ceramide and 4 is Gal-ceramide (42). Basic glycosphingolipids refer only to psychosine or galactosylsphingosine that contains Gal residue, while psychosine sulfate belongs to amphoteric glycosphingolipids. Last, sphingolipids can be covalently attached to proteins to form adducts such as β-hydroxyceramides and inositol-phosphoceramides, which can be found on surface proteins of skin cells and membrane anchor proteins of fungi (43, 44). In cells, ceramides the building blocks of all sphingolipids, can be synthesized *de novo* from palmitoyl-CoA and serine or sphingosine and a fatty acyl CoA. Sphingomyelin contains phosphate, but not the glycerol backbone that glycerophospholipids have. When a glucosyl or a galactosyl group is attached to the C1 of ceramides, cerebrosides are formed. Galactocerebrosides are used to form sulfatides, whereas glucocerebrosides are used to synthesize gangliosides, globosides, and other related compounds (45).

## Distribution of sphingolipids in foods

The contents of sphingolipids in foods vary from low in fruits and some vegetables to high in dairy products, egg and soybeans (8). Table 2 summarizes the distribution and contents of sphingolipids in common foods. Since most sphingolipid research results have been focused on their structures, we could not collect contents of all sphingolipids in food. Based on the reported sphingolipid contents in nmol/g, the estimates in mg/100 g were converted using an average molecular weight for sphingosines of 299 g/mol, sphinganines of 301 g/mol, ceramides of 587 g/mol, sphingomyelin of 751 g/mol, and cerebroside of 779 g/mol. The first three sphingoid bases, sphingosines, sphinganine and phytosphingosine, are usually found in mammals, plants and fungi, and sphingosine (d18:1) is the most abundant of these three in mammalian cells (39, 46). In addition, substantial amounts of C20 and minor amounts of C12 to C22 as well as 1-deoxy and 1-deoxymethyl LCBs can also be found in animal products (47, 48). Phytosphingosines and their derivatives are usually found in plants to form plant sphingolipids, such as phytosphingosine (t18:0) and phytosphingosines 8-enine (t18:1) in potatoes and sweet potatoes, 9-Methyl-4, 8-sphingadienine (d19:2) and 2-Amino-4, 8, 10-octadecatriene-1, 3-diol (d18:3) in rice, mushroom and sea cucumber (49, 50). Many fungi also methylate the alkyl chain at C-9 to produce C19 bases, whereas *Saccharomyces cerevisiae* only produces sphingosine with predominant d18:0, t18:0, and some d20:0, t20:0 (51). Free sphingosine and LCBs are only present in cells at very low levels. Most of them can be phosphorylated to form S1P and other phosphorylated sphingosine bases.

**TABLE 2:** Distribution and contents of sphingolipids in foods^a^.

Content (mg/100 g) dietary sources	Sphingoid bases	Ceramide	Sphingomyelin	Cerebrosides	Gangliosides
**Cereal**					
Rice bran		5.6 (dw) (41)		11.5 (dw) (41)	
Rice (milled)				2.5 (ww) (205)	
Barley				14.4 (ww) (205)	
Corn				11.5 (ww) (205)	
Wheat flour				21.0 (ww) (205)	
Wheat flour				43.0 (dw) (8)	
**Legumes and nuts**					
Full-fat soy flakes	0.8 (dw) (55)	4.9–12.2 (dw) (206)	45.7 (dw) (55)	65.2–309.7 (dw) (206) 11.1–38.3 (dw) (207)	
Defatted soy flakes				19.2–23.9 (dw) (208)	
Almond			304.3 (dw) (209)	6.8 (dw) (210)	
Cashew nut			3.9 (dw) (209)	3.9 (dw) (210)	
Hazelnut			15.7 (dw) (209)	2.1 (dw) (210)	
Pine nut			376.9 (dw) (209)	4.2 (dw) (210)	
Peanut			5.9 (ww) (8)	3.9 (dw) (210)	
Pumpkin seed				3.1 (dw) (210)	
Walnut			612.9 (dw) (209)	2.5 (dw) (210)	
Pecans			373.5 (dw) (209)		
Pistachios			0.4 (dw) (209)		
**Livestock and poultry meat**					
Meat					0.35–1.1 (ww)
Pork			20.3 (ww) (52)		
Ham			17.8 (ww) (52)		
Beef			28.6–69.0 (ww) (41, 52)		
Fat cooked ground beef					1.7 (ww) (211)
Fat raw ground beef					1.6 (ww) (211)
Beef blade steak					1.0 (ww) (212)
Lamb			33.2 (ww) (52)		
Chicken			41.8 (ww) (52)		
Chicken thigh					1.4 (ww) (212)
Chicken breast					1.0 (ww) (212)
Turkey			35.0 (ww) (52)		
**Aquatic products**					
Plaice			9.6 (ww) (52)		
Herring			7.2 (ww) (52)		
Cod			6.5 (ww) (52)		
Turbot		2.9 (ww) (213)	6.4 (ww) (213)	0.3 (ww) (213)	0.9 (ww) (212)
Trout		2.5 (ww) (213)	4.2 (ww) (213)		
Mackerel		5.5 (ww) (213)	3.9 (ww) (213)		
Grass carp		11.5 (ww) (213)	1.7 (ww) (213)	0.3 (ww) (213)	
Shellfish			0.8 (ww) (214)		
Mussel		22.9 (ww) (214)		1.4 (ww) (214)	
Clam		19.3 (ww) (214)		7.4 (ww) (214)	
Scallop		16.7 (ww) (214)		6.8 (ww) (214)	
Oyster		12.1 (ww) (214)		2.2 (ww) (214)	
Island mackerel					6.6 (ww) (212)
King salmon					1.1 (ww) (212)
Snapper					0.8 (ww) (212)
Tuna					0.1 (ww) (211)
Microalgae			250–2760 (dw) (215)	2300 (dw) (215)	
**Egg, milk, and dairy products**					
Chicken egg yolk		4.4 (ww) (216)	190.0 (ww) (216)		1.7 (ww) (211)
Milk		0.1 (ww) (217)	23.4–47.3 (ww) (218)	13.6–175.2 (ww) (218)	0.05 (ww) (211)
Cheese	2.0 (ww) (55)		24.3–365.7 (ww) (218)	12.22–27.9 (ww) (218)	0.07 (ww) (211)
Butter			29.2–44.4 (ww) (218)	18.7–19.9 (ww) (218)	
Non-fat dry milk	43.8 (ww) (55)		15.2 (ww) (55)		
Yogurt	0.4 (ww) (55)		10.4 (ww) (55)		0.07 (ww) (211)
**Vegetables**					
Cauliflower			13.7 (ww) (8)		
Cucumber			2.0 (ww) (8)		
Lettuce			3.8 (ww) (8)		
Tomato			3.1 (ww) (8)		
Potato			5.2 (ww) (8)		
Sweet potato				50 (ww) (8)	
Spinach				5.0 (ww) (8)	
Soybeans				180 (ww) (8)	
**Fruits**					
Orange			1.8 (ww) (8)	4.9 (ww) (205)	
Banana			1.5 (ww) (8)	1.2 (ww) (205)	
Apple				7.1 (ww) (205)	
Grape				7.9 (ww) (205)	
Kiwifruit				6.2 (ww) (205)	
Lemon				4.9 (ww) (205)	
Pear				6.8 (ww) (205)	
Strawberry				3.4 (ww) (205)	

^a^dw means dry weight ratio, ww means wet weight ratio. Based on estimates of reported sphingolipid content (nmol/g), the conversion to mg/100 g was calculated using an average molecular weight for sphingosines of 299 g/mol, sphinganines of 301 g/mol, ceramide of 587 g/mol, sphingomyelin of 751 g/mol, and cerebroside of 779 g/mol.

Ceramides are widely distributed in mammals, especially in the skin (52). Sphingomyelins are high in animal-derived foods, such as aquatic products (2–10% of total phospholipids), meat products (5–10% of total phospholipids), eggs (approximately 1.5% of total phospholipids), and dairy products (approximately 25% of total phospholipids). Bioactive complex lipids are very abundant in milk and can be used as the main raw material for obtaining sphingomyelin and gangliosides (53). Insects mainly contain ceramide phosphate ethanolamine, and fungi have ceramide phosphate inositol and mannose-containing head group, and not found in most plants except legumes and nuts (2, 54). Soybeans are a good source of the plant-derived sphingolipids such as full-fat soy flakes (55). The types of fatty acids in sphingomyelins are different in foods. Taking eggs and milk for example, sphingomyelins derived from eggs have higher percentage of short-chain saturated fatty acids (SFAs) than that from milk (56). Although the content of glycosphingolipids in foods are lower than that of sphingomyelins, they are widely distributed in cereal, legumes and nuts, livestock and poultry meat, aquatic products, egg, milk and dairy products, vegetables, and fruits, as shown in Table 2. The sphingolipids in vegetables and fruits are relatively lower than other foods, mainly containing cerebrosides or glycosyl inositol phosphate ceramides (GIPCs) which can connect complex sugar chains (57, 58). Glucosylceramide-based sphingolipids, which have the basic structure of the tetrasaccharide ceramide series, are the main glycosphingolipids in plants, whereas some mannosylceramide series also exist (59). In invertebrates and vertebrates, different saccharides and their derivatives are added to the first sugar moiety of cerebroside, which is mainly glucose, to form hundreds of gangliosides with different head group structures. In addition, vertebrates also have a series of glycolipids containing galactose (60).

## Metabolism of sphingolipids

### Metabolic pathways of sphingolipids

**FIGURE 1:**
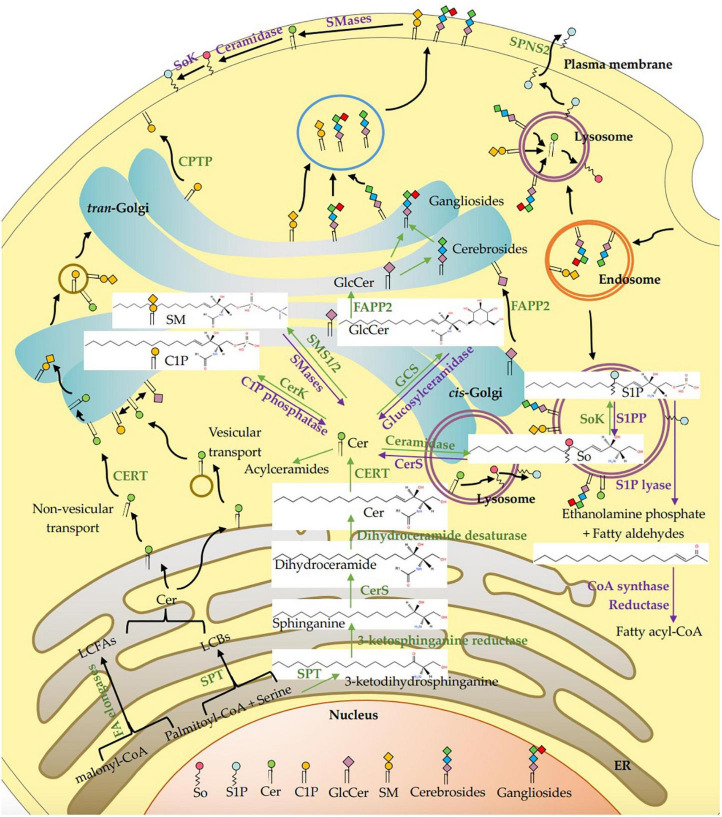
Intracellular metabolism and transport of sphingolipids in mammalian cells. The *de novo* synthesis of sphingolipids initiates with the synthesis of ceramide (Cer) through condensation by SPT complex utilizing palmitoyl-CoA and serine, which is followed by reduction, acylation, and desaturation on the cytosolic leaflet of the ER. Fatty acids with 12–16 carbons are extended by a family of elongase enzymes to form long-chain fatty acids (LCFAs, C18-C26) in the ER, which are used in the synthesis of Cer. Cers are transported to the Golgi apparatus through vesicular transport, and non-vesicular transport via the Cer transport protein (CERT). A variety of head groups are added to Cer to form complex sphingolipids in the Golgi apparatus such as sphingomyelin (SM), ceramide-1-phosphate (CIP), and glucosylceramide (GlcCer) by sphingomyelin synthase 1/2 (SMS1/2), ceramide kinase (CerK) and glucosylceramide synthase (GCS), respectively. The GlcCer is then translocated into the lumen of the Golgi apparatus via a membrane-bound transporter and further transformed into cerebrosides and gangliosides by different enzymes. C1P is transferred from the Golgi apparatus to plasma membrane via non-vesicular translocation of C1P transfer protein (CPTP). The SM, cerebrosides and gangliosides are delivered to the plasma membrane via vesicular trafficking, and then SM is metabolized to Cer, sphingosine (So) and sphingosine-1-phosphate (S1P). Cer is deacylated by ceramidase to generate So, and then phosphorylated by sphingosine kinase (SoK) to produce S1P in the lysosome and also in plasma membrane. The S1P lyase cleaves S1P into ethanolamine phosphate and fatty aldehydes, the latter are further metabolized and reduced to fatty acyl-CoA. All synthetic reactions to produce complex sphingolipids from Cer are reversible, such as SM, C1P, and GlcCer are hydrolyzed by sphingomyelinase (SMase), C1P phosphatase and glucosylceramidase, respectively. S1P is dephosphorylate by S1P phosphatase (S1PP) to get So, which is acylated by ceramide synthases (CerS) to form Cers. In the lysosome, SM, cerebrosides and gangliosides are metabolized by each hydrolase to produce Cer, and then degraded by acid ceramidase to generate sphingosine. In addition, Cers can also be acylated at the 1-OH position to form acylceramides. ER, Endoplasmic reticulum; SPT, serine palmitoyltransferase; CerS, ceramide synthase; CERT, ceramide transfer protein; Cer, ceramide; LCFAs, long chain fatty acids; LCBs, long chain bases; CerK, ceramide kinase; C1P, ceramide-1-phosphate; SMS1/2, sphingomyelin synthase; SMases, Sphingomyelinase; CerS, ceramide synthase; So, sphingosine; SoK, sphingosine kinase; S1P, sphingosine-1-phosphate; S1PP, sphingosine-1-phosphate phosphatase; GlcCer, glucosylceramide; GCS, glucosylceramide synthase; FAPP2, phosphatidylinositol-4-phosphate adaptor protein 2.

In mammals, about 40 enzymes catalyze *de novo* synthesis, catabolism, recycling and interconversion of sphingolipids and products derived from them as summarized in Figure 1. The *de novo* synthesis of sphingolipids begins with the ceramide production. In general, 3-ketodihydrosphinganine is formed after the rate-limiting enzyme serine palmitoyltransferase (SPT) complex condenses palmitoyl-CoA and serine in a reaction that requires pyridoxal phosphate, NADPH, and Mn^2+^ (61, 62), and then reduced by 3-ketodihydrosphingosine reductase to generate sphinganine (47, 61). The mutations of the genes encoding SPTLC1 and SPTLC2, the key subunits of SPT, induce a permanent shift in the substrate specificity from L-serine to L-alanine and L-glycine, which results in the formations of atypical and neurotoxic metabolites, the 1-deoxy-sphingolipids and 1-deoxymethyl sphingoid bases, respectively, and their acylated ceramides (63). Besides, the recently discovered SPTLC3 prefers myristic acid to palmitic acid (64) and may use stearic acid as a fatty acid substrate (65). SPT can also use alanine and glycine, as well as myristate and stearate, to produce a large amount of sphingosine bases. After that, the sphinganine is acylated by one of six ceramide synthases (CerS1-6) to form dihydroceramide (66), which is desaturated by dihydroceramide desaturase to generate ceramide (67). The other fatty acyl moiety in sphingolipids is long chain fatty acids (LCFAs) with 18–26 carbons, which are formed by a family of elongases extending myristic acid and palmitic acid in the ER (68). These variations in LCBs are combined with different acyl groups by the actions of CerS1-6, which makes ceramides a family of closely related but distinct molecules with different functions owing to variations in LCFA length and hydroxylation and desaturation of the LCB and LCFA components (69).

Ceramides are transported to the Golgi apparatus via vesicular and non-vesicular mechanisms (11). The latter mechanism *via* a ceramide transport protein (CERT) to assemble into sphingomyelin only exists in mammals (70), as shown in Figure 1. A variety of head groups are added to ceramide to form complex sphingolipids in the Golgi apparatus such as sphingomyelin, CIP, inositol phosphorylceramides and glycosphingolipids. The sphingomyelin synthase 1/2 (SMS1/2) enzymes located in the luminal side of the Golgi membrane transfer a phosphocholine headgroup from phosphatidylcholine to ceramide yielding diacylglycerol and sphingomyelin (71). According to Mitsutake and Igarashi, sphingomyelin synthase SMS2 on the plasma membrane dynamically regulates the activity of lipid microdomains and mice lacking SMS2 are resistant to the effects of a high-fat diet (HFD) on body weight, glucose intolerance, and fatty liver (72). Galactosylceramide (GalCer) and glucosylceramide (GlcCer) are synthesized by ceramide galactosyltransferase (CGT) and glucosylceramide synthase (GCS) transferring the monosaccharides from UDP-galactose and UDP-glucose to ceramide at the cytoplasmic side of the Golgi membrane, respectively (61, 73). The GlcCer is then translocated into the lumen of the Golgi *via* a membrane-bound transporter and further transformed to lactosylceramide (LacCer), which is synthesized by LacCer synthase transferring galactose from UDP-galactose to GlcCer (41, 74). The LacCer acts as a common backbone to produce more complex glycosphingolipids by various enzymes (41). Phosphatidylinositol-4-phosphate adaptor protein 2 (FAPP2) can transfer GlcCer among Golgi networks and couple it specifically to the synthesis of cerebrosides instead of anionic gangliosides (75). The sphingomyelin, cerebrosides and gangliosides are delivered to the plasma membrane *via* vesicular trafficking (76). Sphingomyelin and glycosphingolipids are delivered to the plasma membrane *via* vesicular transport, while C1P is transferred from the Golgi to other compartments such as the plasma membrane *via* non-vesicular translocation activity of C1P transfer protein (CPTP). On the cell membrane, sphingomyelin can be metabolized to ceramide on the outer leaflet of the membrane, where it is hydrolyzed to sphingosine by ceramidase, and metabolized to S1P by sphingosine kinase (SoK) (77).

Ceramides can be phosphorylated by ceramide kinase (CerK) to generate C1P, which can be dephosphorylated by a C1P phosphatase (78). Ceramides can also be acylated at the 1-OH position to form 1-O-acyl-ceramides (79). In addition, recent studies have shown that the ω-acylation of ceramides forms three-chained ceramides in the skin, thereby regulating epithelial permeability and skin barrier function (80). Ceramides can also be deacylated to generate sphingosine by ceramidase, and then phosphorylated by SoK to produce S1P in the lysosome and on plasma membrane (8). The S1P on the plasma membrane is flipped through the plasma membrane and transported to exocytoplasmic leaflets by the spinster homolog 2 (SPNS2), where it interacts with its receptors (81). The S1P lyase cleaves S1P (or dihydro S1P) into ethanolamine phosphate and fatty aldehydes, the latter are further metabolized and reduced to form acyl-CoA (76). All synthetic reactions to produce complex sphingolipids from ceramides are reversible. For instance, sphingomyelin, C1P and GlcCer are hydrolyzed by sphingomyelinase (SMase), C1P phosphatase and glucosylceramidase, respectively. This pathway may rapidly increase intracellular ceramide levels, while S1P catabolism is irreversible (82). S1P is catalyzed by S1P phosphatase (S1PP) and S1P lyase to dephosphorylate to sphingosine or irreversibly cleave to ethanolamine-1-phosphate and hexadecenal, respectively (83). The sphingolipids may be recirculated through endocytosis and transported from the endosome to the lysosome. In the lysosome, sphingomyelin, cerebrosides and gangliosides are metabolized to ceramides by hydrolases, and the ceramides are then degraded by acid ceramidase to generate sphingosine. Due to its positive charge, sphingosine is able to leave the lysosome and moves among the membranes such as ER membrane to be recycled (84). The sphingolipids have diverse biological activities in a receptor-dependent or -independent manner. Ceramide and sphingosines are mainly involved in cell apoptosis (85, 86), while S1P and C1P are related to cell survival (87), suggesting their antagonistic effects in some cases. Since ceramide and diacylglycerol may have opposite effects on cell proliferation and survival, SMS1/2 probably play an important role in regulating cellular fate. Therefore, the enzymes involved in these metabolic pathways maintain the homeostasis of bioactive sphingolipid metabolites.

### Dietary factors affecting the metabolism of sphingolipids

Many prospective studies have shown that different dietary patterns affect *de novo* sphingolipids synthesis, which in turn alters sphingolipid metabolism in human. The feeding of a HFD stimulates *de novo* synthesis of sphingolipids and alter sphingolipid metabolism in cells and tissues, which is associated systemic insulin resistance and imbalance of lipid accumulation, thereby exacerbating obesity-related diseases. HFD enhances *de novo* synthesis and turnover of sphingolipids through the salvage pathway, which promotes the synthesis and accumulation of ceramides, more specifically long chain ceramide species in skeletal muscle, plasma and liver tissues (88–90). Intake of SFAs enriched HFD can upregulate genes involved in ceramide synthesis (16), and promote releases of inflammatory cytokines such as tumor necrosis factor-α (TNF-α) and interleukin-1β (IL-1β) by inhibiting protein kinase B (Akt/PKB) (91). In addition, HFD can also increase the levels of sphingomyelin and S1P in the liver, skeletal muscle, adipose tissue, and cardiovascular tissue (89). Plant foods-based Mediterranean diet (92) has been negatively associated with the risks of cardiovascular diseases (CVDs) (93). This is associated with decrease in the plasma ceramide concentration. This may be attributed to the high ratio of monounsaturated fatty acids (MUFAs) to SFAs in the Mediterranean diet, which can inhibit the SFAs-induced accumulation of ceramides (17). In contrast, EPA (C20:5, n-3) and DHA (C22:6, n-3) decrease the expressions of genes involved in ceramide synthesis and ceramide content in the skeletal muscle and liver in rodents (88). Vitamins B6, C, D, and K have been shown to regulate sphingolipids metabolism (18–23). S1P lyase is a vitamin B6-dependent enzyme that can degrade S1P in the last step of sphingolipid metabolism. A genetic disease called SPL dysfunction syndrome (SPLIS) is caused by mutations in SGPL1, which encodes sphingosine phosphorylase (SPL). SPLIS patients exhibit lymphopenia, nephropathy, adrenal insufficiency, and/or neurological deficits. Vitamin B6 supplementation increases S1P abundance and activity levels, and decreases sphingolipids in the SPLIS patients (18). In addition, SPT responsible for 3-ketodihydrosphinganine synthesis is also a vitamin B6-dependent enzyme (19). One study has shown that the treatment with 1.2 mM calcium and 50 μg/mL vitamin C can significantly stimulate the content of ceramide in human keratinocytes (20). There may also be a relationship between the vitamin D intake and endogenous sphingolipids concentration in humans. Vitamin D supplementation can change levels of long-chain ceramides in the circulation and related sphingolipids metabolism, especially the increased levels of ceramide (C18) and dihydroceramide (C18) in subjects with type 2 diabetes (T2DM) (21). γ-tocotrienol, a form of vitamin E, can increase the levels of dihydroceramide and sphinganine but does not affect ceramide and sphingosine in cells (22). Vitamin K can regulate sphingolipids metabolism in the nervous system (23). However, more *in vivo* studies are needed to understand how vitamins regulate sphingolipid metabolism.

## Absorption and utilization of dietary sphingomyelin

### Pathways for the digestion and absorption of dietary sphingomyelin

The digestion and utilization of dietary sphingolipids starts in the jejunum section of the small intestine. The typical plasma membrane of mammalian cells contains about 20% cholesterol, 15–20% sphingomyelin, and 5% glycolipids of the total lipids, whereas the small intestinal brush border contains about 10% cholesterol, 5% sphingomyelin and over 30% glycolipids of the total lipids (7, 94). Bouhours and Guignard detected 750 nmol of sphingomyelin, 160 nmol of free ceramide, and 390 nmol of glucosylceramide from 1 mL of isolated rat intestinal epithelial cells (95). The levels of these sphingolipids change with the differentiation of rat epithelial cells and the development of the intestine (96, 97). Humans normally consume 2–8 g/day of phospholipids from the Western diet, accounting for 1–10% of total daily fat intake (98). Among them, sphingomyelin is about 0.3–0.4 g/day, which is mainly derived from fish, meat, milk and egg products. After digestion, about 3–6 g/day of phospholipids enter the blood through lipoproteins, of which sphingomyelin accounts for about 7%. In addition, sphingomyelin in human bile accounts for about 2% of total phospholipids, and about 0.1–0.2 g/day mainly palmitoyl and stearoyl species is delivered to the intestines (14).

**FIGURE 2:**
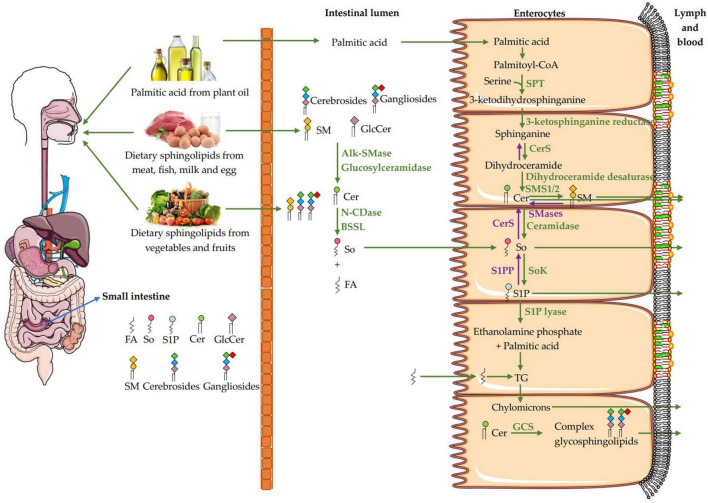
Pathways for the digestion and absorption of dietary sphingomyelin. The digestion of dietary sphingomyelin (SM) in the small intestinal lumen begins with the decomposition of SM to ceramide by Alk-SMase. Ceramide is further hydrolyzed into sphingosine (So) and fatty acids by N-CDase and BSSL, then the So is absorbed intact into the enterocytes. In the enterocytes, part of So comes from the *de novo* synthesis of palmitic acid and serine, and part of it comes from intestinal absorption. When the So is absorbed into the enterocytes, only a small amount of free So is transported with chylomicrons to the lymph and blood. Most of the So is catalyzed by sphingosine kinase (SoK) to generate sphingosine-1-phosphate (S1P), and then degraded to ethanolamine phosphate and hexadecenal by S1P lyase, or to a less extend dephosphorylated to new So by S1P phosphatase (S1PP) and then to form ceramides and sphingomyelin. Hexadecenal will be converted to palmitic acid and then esterified into triglycerides (TG), which can be incorporated into chylomicrons for transport. FA, fatty acid; So, sphingosine; S1P, sphingosine-1-phosphate; Cer, ceramide; GlcCer, glucosylceramide; SM, sphingomyelin; SMases, Sphingomyelinase; Alk-SMase, alkaline SMase; N-CDase, neutral ceramidase; BSSL, bile salt-stimulated lipase; SPT, serine palmitoyltransferase; CerS, ceramide synthase; SMS1/2, sphingomyelin synthase 1/2; CerS, ceramide synthase; SoK, sphingosine kinase; S1PP, sphingosine-1-phosphate phosphatase; TG, triglycerides; GCS, glucosylceramide synthase.

Figure 2 shows the digestion and absorption of dietary sphingomyelin. After the dietary lipids are digested in gastrointestinal tract, triglyceride (TG) is hydrolyzed into free fatty acids, n-2 monoglycerides and a small amount of diacylglycerides by pancreatic lipase. Cholesterol esters are hydrolyzed by cholesterol esterase to form free cholesterol and fatty acids. Glycerophospholipids are cleaved by pancreatic phospholipase at n-1 or n-2 position to produce lysophospholipids and free fatty acids. Sphingomyelin is hydrolyzed to ceramide and phosphorylcholine by Alk-SMase in the intestinal mucosa under the optimal pH of 9.0 (99). Alk-SMase is produced by the liver and released into the jejunum vie bile, and is activated in the presence of bile salts and trypsin (3). Alk-SMase, with the hundred times of hydrolysis capacity higher than that of neutral SMase and acidic SMase, is strictly bile salt dependent and effectively stimulated by taurocholate and taurochenodeoxycholate (14, 100). Most sphingomyelin and its hydrolysate ceramides could not be absorbed intact and contribute to the chylomicron and plasma sphingomyelin pools (14). Ceramides are further hydrolyzed into sphingosine and fatty acids by intestinal N-CDase and bile salt-stimulated lipase (BSSL), while sphingosine could be absorbed into intestinal mucosal cells and rapidly metabolized in the enterocytes (13). BSSL from pancreatic juice plays a key role in the digestion of TG and hydrolysis of the amide bond of ceramide between sphingosine and fatty acyl group. The activity of BSSL depends on bile salts with the optimal pH 8.5. It is worth noting that there is almost no ceramide formation in the proximal intestine where BSSL is most active (101, 102). All the digested products and bile acid (BA) can be assembled into micelles, which assist in crossing the unstirred water layer for entering the enterocytes.

When sphingosine is absorbed into the enterocytes, only a small amount of free sphingosine is incorporated into chylomicrons to be transported in the lymph and then, the blood. Most of the sphingosine receives a phosphate to generate S1P in the enterocytes, a process catalyzed by SoK. S1P is mainly degraded to ethanolamine phosphate and hexadecenal by S1P lyase, or to a less extend dephosphorylated to new sphingosine by S1PP and then to form ceramides and sphingomyelin. Fatty acids can be esterified into TG, and loaded with phospholipids and cholesterol esters onto chylomicrons, which enter the blood through lymphatic system (3, 14). Interestingly, the sphingosine first can be dephosphorylated by Sok to form S1P and secondly phosphorylated by S1PP to generate the new sphingosine, then acylated by CerS to form ceramide and more complex sphingolipids such as sphingomyelin and glycosphingolipids, which can be then incorporated into chylomicrons in the enterocytes for the release into the lymph. Dephosphorylation and then phosphorylation are prerequisites for the synthesis of sphingolipids by exogenous sphingosine in the enterocytes (13–15). In addition, dietary glycosphingolipids such as glucosylceramides, cerebrosides and gangliosides are degraded to produce ceramides by a series of enzymes such as glucosidase, galactosidase, glucosylceramidase, galactosylceramidase (3, 41). In the enterocytes, ceramides can also be synthesized from dietary palmitic acid and serine as shown in Figure 1.

### Transport of sphingomyelin in plasma lipoproteins

Lipoproteins can be divided into chylomicrons, very low-density lipoproteins (VLDL), intermediate density lipoproteins (IDL), and low-density lipoproteins (LDL) and HDL according to their densities. HDL are further categorized into HDL_1_, HDL_2_, HDL_3_ based on their densities. HDL_1_ only appears after taking a high-cholesterol diet. Abnormal lipoprotein metabolism is associated with arteriosclerosis, diabetes, obesity and tumor occurrence (103). The concentrations of more than 200 sphingolipids in plasma lipoproteins vary. Sphingomyelin, LacCer, hexosylceramide, ceramides, S1P and dhS1P, C1P, sphingosine and dihydrosphingosine, are constituted 87.7, 5.8, 3.4, 2.8, 0.22, 0.15, and 0.005% of total sphingolipids in the human blood, respectively (104). Sphingolipids in lipoproteins can be secreted with apolipoprotein B (apoB) or apoA-I, and can be transferred from one lipoprotein to another. In addition, the complex sphingolipids in the lipoproteins can be modified from other existing sphingolipids, or decomposed into other sphingolipid components, such as ceramides, S1P, and Glc-ceramide (103). The human plasma contains 416 mg/ml sphingomyelin, which accounts for about 20% of total plasma phospholipids (104, 105) and 87.7% of total plasma sphingolipids (104, 105). The C_16_ and C_24:1_-sphingomyelin are the major species in human plasma. Approximately 3.15, 32.85, 34.1, and 29.9% plasma sphingomyelin are present in VLDL, LDL, HDL_2_, and HDL_3_, respectively. The particle concentrations of these four lipoproteins in human plasma are 0.073, 1.514, 8.35, and 22.1 nmol/ml, and their molecular weights are 10.0 × 10^6^, 2.0 × 10^6^, 4.0 × 10^5^, and 2.0 × 10^5^, and percentages of protein composition are 10, 20, 40, and 55%, respectively (104). However, the amount of sphingomyelin has been significantly reduced in HDL and increased in LDL and VLDL in women with insulin-dependent diabetes (106). Approximately 75% of plasma sphingomyelin is present in LDL and VLDL, and about 25–37% is present in HDL, showing that the decrease of sphingomyelin, phosphatidylcholine and lysophosphatidylcholine may be potentially atherogenic. The reduction of phospholipid content in HDL may weaken its ability to promote the outflow of cholesterol from cells, and inhibit the transfer of cholesterol esters from HDL to larger lipoproteins containing apoB (106). Approximately 8.73, 39.94, 28.74, and 22.59% plasma ceramides are present in VLDL, LDL, HDL_2_, and HDL_3_, respectively. The prominent ceramide is C_24_-ceramide in human plasma. About 1.33, 3.73, 16.3, and 78.64% plasma S1P are present in VLDL, LDL, HDL_2_, and HDL_3_, respectively. The ratios of sphingomyelin/ceramides in VLDL, LDL, HDL_2_, and HDL_3_ are 22.3:1, 50.9:1, 72.9:1, and 78.9:1,whereas the ratios of ceramides/S1P in those lipoproteins are 49.8:1, 80.7:1, 13.4:1, and 2.2:1, respectively (104). In addition, more than 50 species of complex glycosphingolipids account for about 9–10% of plasma sphingolipids, of which the most abundant ones are GlcCer and LacCer, accounting for about 8–14, 46–60, and 28–44% in VLDL, LDL, and HDL, respectively (103, 104).

**FIGURE 3:**
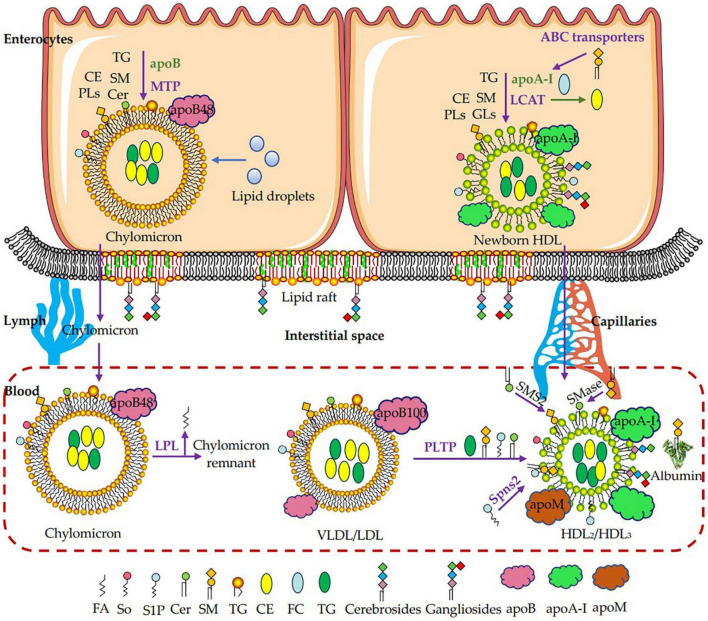
Transport of dietary sphingomyelin from enterocytes to plasma in lipoproteins. Most sphingomyelin (SM) and ceramide (Cer) can be reformed from sphingosine (So) in enterocytes, which will be transferred to the nucleation site with triglyceride (TG), free cholesterol (FC), cholesterol esters (CE) and other phospholipids (PLs) under the action of MTP and incorporated onto apolipoprotein B-48 (apoB-48) to form the original lipoprotein. The original lipoprotein then fuses with the lipid droplets to form chylomicron, which is secreted into the lymph and blood. TG in chylomicrons is hydrolyzed into free fatty acids by lipoprotein lipase (LPL), which leads to the formation of chylomicron residues in the circulation. Apolipoprotein A-I (apoA-I) interacts with the ATP binding cassette (ABC) transporters family and accepts SM and glycosphingolipids to form pre-β HDL. Then lecithin–cholesterol acyltransferase (LCAT) converts FC to CE in pre-β HDL, which moves to the core of the newborn HDL and is secreted into the blood to form mature HDL_2_ and HDL_3_. In the plasma membrane, synthesis of SM by SMS2 from Cer can contribute to SM in HDL, and degradation of SM by SMase may also increase Cer in HDL. S1P from the plasma membrane of peripheral tissues such as erythrocytes and platelets, is delivered to HDL with apolipoprotein M (apoM) and apoA-I *via* Spinster2 (Spns2). In addition, SM, Cer, S1P and TG are transferred from VLDL/LDL to HDL *via* phospholipid transfer protein (PLTP). FA, fatty acid; So, sphingosine; S1P, sphingosine-1-phosphate; Cer, ceramide; SM, sphingomyelin; PLs, phospholipids; CE, cholesterol esters; FC, free cholesterol; TG, triglyceride; apoB, apolipoprotein B; apoA-I, apolipoprotein A-I; apoM, apolipoprotein M; MTP, microsomal triglyceride transfer protein; LPL, lipoprotein lipase; GLs, glycosphingolipids; LCAT, lecithin–cholesterol acyltransferase; ABC transporters, ATP binding cassette transporters; SMases, Sphingomyelinase; SMS2, sphingomyelin synthase 2; PLTP, phospholipid transfer protein; Spns2, Spinster2.

Sphingomyelin formed in the enterocytes enters the body *via* chylomicrons as shown in Figure 3. The absorption of sphingosine leads to the formation of sphingomyelin and ceramides, which are incorporated into pre-chylomicron with TG, free cholesterol, cholesterol esters and other phospholipids under the action of microsomal triglyceride transfer protein (MTP) with apoB-48 in enterocytes. The pre-chylomicron then fuses with the lipid droplets to form chylomicron, which is secreted into the lymph circulation and then the blood circulation. TG in chylomicron is hydrolyzed into free fatty acids by lipoprotein lipase (LPL), which leads to the formation of chylomicron remnants in the circulation (14, 103, 107). A part of sphingomyelin may also associate with apoA-I in the Golgi and be assembled into nascent HDL that is then secreted to the plasma and is responsible for reverse cholesterol transport to move cholesterol from extrahepatic tissues to the liver. New born HDL is mainly synthesized in the liver and can also be synthesized in the small intestine (14). The blood of healthy people mainly contains HDL_2_ and HDL_3_. ApoA-I interacts with the ATP binding cassette (ABC) transporters family and accepts sphingomyelin and glycosphingolipids to form pre-β HDL. Then lecithin–cholesterol acyltransferase (LCAT) converts free cholesterol to cholesterol esters in pre-β HDL, which move to the core of the newborn HDL and migrate with the lipoprotein particle in the blood to form mature HDL_2_ and HDL_3_ (103). *In vitro* experiments show that ABCA1, ABCA7, or ABCG1 expressed in HEK293 cells incubated with apoA-I efflux sphingomyelin (108–110). There are few studies on the transport of glycosphingolipids. Unlike sphingomyelin and ceramides, glycosphingolipids cannot be transported to apoB-containing lipoproteins by MTP. Instead, it may be assembled into HDL under the action of two possible transporters ABCA12 and ABCC1. Studies have shown that ABCC1 and ABCA12 are involved in the transport of glucosylceramide *in vivo*, but it is unclear whether its effluxes glucosylceramide to HDL or apoA1, or plays a role in plasma glycosphingolipid transport or metabolic disorders (103, 111, 112). Therefore, more *in vivo* evidence is needed to show that these transporters in basal levels play a role in sphingomyelin release, since their overexpression may limit the fluidity of membrane, which is conducive to outflow. As for ceramides, how they are involved in the assembly of HDL is still unknown. Beside in the enterocytes, ABC transporters on the plasma membrane of macrophages, liver or other peripheral tissues can efflux sphingomyelin to HDL, apolipoprotein such as apoA-I, albumin or other receptors (103). Phospholipid transfer protein (PLTP) can maintain particle stability during the wide range of lipoprotein particle modifications in plasma. It can transfer sphingomyelin, ceramides, S1P and TG from VLDL/LDL to HDL (14). However, it is not clear whether PLTP can transfer GluCer, GalCer or other complex sphingolipids. On the plasma membrane, synthesis of sphingomyelin by SMS2 from ceramides can contribute to HDL sphingomyelin, degradation of sphingomyelin by SMase may also increase HDL ceramides (103). In addition, S1P from the plasma membrane of peripheral tissues such as erythrocytes and platelets, can be delivered to HDL with apoM and apoA-I *via* Spinster2 (Spns2) (103, 113). How other sphingolipids components are incorporated into lipoprotein particles is unclear so far, and more research is needed to evaluate these and other possibilities.

## Functions of dietary sphingomyelin in chronic metabolic diseases

### Effects of dietary sphingomyelin on lipid profile

Metabolic syndrome (MetS) has become a global public health issue. It is the aggregate of pathological conditions in which proteins, fats, carbohydrates and other substances are metabolized abnormally, leading to central and abdominal obesity, insulin resistance, atherogenic dyslipidemia and systemic hypertension (114). Genetic defects of genes involved in metabolism may also lead to MetS. Additionally, dysfunctions of the liver or pancreas might result in MetS (115). Individual therapy of hypertriglyceridemia, hyperglycemia, and hypertension, as well as dietary restriction and frequent exercise, have been suggested as treatments for MetS. The dietary sphingomyelin and its metabolites may play an important role in regulating cholesterol homeostasis and lipid metabolism, and alleviating the symptoms of obesity, diabetes and atherosclerosis as shown in Table 3. Previous studies have shown that dietary sphingomyelin and its metabolites can significantly modify the plasma and hepatic cholesterol and TG metabolism in rats (116, 117) and inhibits the absorption of cholesterol both in Caco-2 cells and animal studies (118–120). Interestingly, sphingomyelin from milk is more effective in inhibiting cholesterol absorption than that from eggs, which may be attributed to the higher degree of saturation and longer chain length of fatty acyl group in milk sphingomyelin (56). Natural phospholipids, especially sphingomyelin, have high affinity for cholesterol to slow the rate of luminal hydrolysis, micellar solubilization, and transfer of micellar lipids to the enterocytes (121). We have found that the ability of egg sphingomyelin to inhibit cholesterol absorption, and simulate cholesterol transport at a higher rate than phosphatidylcholine in the Caco-2 monolayer. This is mediated by downregulation of the Niemann-Pick-Like Protein 1 (NPC1L1) mRNA, which is a key player in cholesterol absorption (122). A randomized crossover study shows for the first time that diets enriched with milk sphingomyelin, increased serum HDL cholesterol levels, but did not affect the serum TG, total cholesterol (TC), non-HDL cholesterol levels, also cholesterol absorption and cholesterol fractional synthesis rate in ten healthy adult males and females (123). The milk sphingomyelin also could regulate intestinal permeability and affect the intestinal tight junction protein expression through increasing the IL-8 secretion in Caco-2/TC7 cells, which may provide a clue to the protective role of obesity or leaky gut diseases (124). Sphingosine treatment significantly reduces cholesterol absorption in Caco-2 and HT-29-D4 cells (125). Due to the differences of sphingoid bases and fatty acid compositions, dietary sphingomyelin can improve lipids absorption and metabolism, probably *via* micellar solubilization, affinity to the hydrolases, or regulation of key proteins involved in cholesterol absorption.

**TABLE 3:** Effects of dietary sphingomyelin and its metabolites on human physiology and chronic metabolic diseases.

Metabolic disorders	SM and its’ metabolites	Model	Treatment and duration	Results	References
Normal physiology	SLs from ox brain^a^	ExHC rats	0.5 and 2% (wt/wt) SLs for 2 weeks	^c^↓ Serum TG and liver CE levels; ↑ serum SM and liver PLs levels — Liver TG and serum CE levels	(116)
	SLs from bovine brain^b^	Donryu rats at 4 weeks old	1% (wt/wt) SLs during two generations	↓ Plasma TC levels; ↑ liver TG levels — Liver TC and plasma TG levels, tissue SLs and PLs levels	(117)
	Milk SM	SD rat	6.5–25 μmol/L ^3^H-SM and ^14^C-cholesterol for 4 h	↓ Cholesterol absorption	(118)
	Milk SM	Caco-2 cells C57L/J mice at 7 weeks old	2% (wt/wt) SM for 4 days	↓ Uptake and esterification of cholesterol in Caco-2 cells ↓ Cholesterol absorption in mice	(119)
	Egg yolk SM	SD rat at 8 weeks old	5 or 10 μmol/h SM and ^14^C-cholesterol for 8 h	↓ Lymphatic absorption of cholesterol, α-tocopherol and fatty acid in a dose dependent manner	(120)
	Milk SM and egg yolk SM	SD rat at 8 weeks old	80 μmol/h SM and ^14^C-cholesterol for 8 h	↓ Lymphatic absorption of cholesterol, fat, and other lipids Milk SM is more is more effective in inhibiting cholesterol absorption than egg SM	(121)
	Egg yolk SM	Caco-2 monolayer	0.6 mmol/L and ^3^H-cholesterol for 2 h	↓ Cholesterol transport in a dose dependent manner alter the physicochemical properties of mixed micelles ↓ NPC1L1, Caveolin 1, SREBP-1/2 mRNA expression	(122)
	Milk SM	Ten healthy adult males and females (ages 18–45)	a randomized crossover study with 4-week washout period 1 g/day milk SM for 14 days	— Serum TG, TC, non-HDL cholesterol levels — Cholesterol absorption and cholesterol fractional synthesis rate — Intraluminal cholesterol solubilization ↑ HDL cholesterol levels	(123)
	So	Caco-2 and HT-29-D4 cells	0–50 μmol/L So and ^3^H-cholesterol for 4 h	↓ Cholesterol uptake in a dose dependent manner ↓ NPC1L1 mRNA expression	(125)
Obesity	Chicken skin SM	Zucker rats fed with HFD at 6 weeks old	0.5 and 2% (wt/wt) SM for 45 days	↓ Hepatic lipid, plasma TC and insulin levels ↑ Adipor2, PPARR and Pdk4 mRNA and ↓Scd1 mRNA	(133)
	Egg yolk SM	C57BL/6 mice fed with HFD	0.3, 0.6, or 1.2% SM (wt/wt) with high-fat diet for 4 weeks	↓ Intestinal cholesterol absorption ↓ Total liver lipids (TC, TG) *via* the LXR-SREBP-1c pathway ↑ Fecal lipid and cholesterol output	(28)
	Milk SM Egg yolk SM	C57BL/6 mice fed with HFD at 8 weeks old	0.1% (wt/wt) milk or egg yolk SM for 10 weeks	↓ Body weight, serum TC, fasting glucose and liver TC, TG levels ↓ PPARγ-related mRNA expression (Scd1, Pparg2, Cd36, Fabp4, Ccl2) ↓ Epididymal adipose tissue inflammation and skeletal muscle lipid accumulation	(134)
	Milk SM Egg yolk SM	C57BL/6 mice fed with HFD at 8 weeks old	0.25% (wt/wt) milk or egg yolk SM for 4 weeks	↓ Body weight, serum TC, and liver TG *via* lipid absorption and metabolism regulation ↓ Serum LPS *via* bifidogenic effects and alterations in distal gut microbiota	(129)
	MPL with 25% SM	C57BL/6 mice fed with HFD at 8 weeks old	1.1 or 1.6% (wt/wt) MPL for 8 weeks	↓ Body weight gain – Plasma markers of inflammation 1.6% (wt/wt) MPL: ↓ hepatic gene expression of macrophage marker F4/80 and ↑ *Bifidobacterium animalis* in cecal microbiota 1.1% (wt/wt) MPL: ↓ *Lactobacillus reuteri* in cecal microbiota	(135)
	Milk SM and Cer	KK-A_*y*_ mice at 3 weeks old	1.7% (wt/wt) of LC-BS (0.13% SM; 0.18% Cer) or 0.5% (wt/wt) Cer-fraction (0.34% Cer) or 0.5% (wt/wt) SM-fraction (0.25% SM) for 4 weeks	LC-BS: ↓ plasma TC and LDL cholesterol and liver TG levels Cer-fraction: ↓ liver TC and TG levels ↓ Stearoyl-CoA desaturase-1 SM-fraction: –plasma and liver lipids	(136)
Diabetes	S1P	HIT-T 15 cells pancreatic islets from ICR mouse at 8 weeks old	10 μmol/L S1P	↑ Insulin secretion from isolated mouse islets ↓ Intracellular cyclic AMP levels in a dose dependent manner	(139)
	S1P	INS-1 cells pancreatic islets from SD rats	100–400 nmol/L S1P or dihydro-S1P for 4.5–5 h	↓ TUNEL analysis and caspase-3 activity ↑ PKC activity ↓ Cytochrome c release ↓ Nitric oxide synthase	(140)
	Milk SM	Obese/diabetic KK-A^y^ mice at 4 weeks old	1% (wt/wt) milk SM for 4 weeks	↓ Serum TC, non-HDL cholesterol, PLs and LDL-cholesterol levels; liver TG, TC, neutral and total lipids levels – Serum neutral lipids and cholesterol levels ↑ Fecal lipids (total lipids, TC, total bile acid and PLs) ↑ SREBP-2, HMG-CoA, and Cyp7a1 mRNA expression ↓ Scd1, Elovl2, Elovl5, Fads2 mRNA expression	(29)
	Sea cucumber Cer and GlcCer	SD rat fed with high-fructose-diet	0.16 g/kg Cer and 0.21 g/kg GlcCer of diet for 6 weeks	↓ Serum glucose levels and glycosylated hemoglobin ↓ Hypertension Cer ↑ glycogen levels, glycogen synthesis and insulin signal transduction in skeletal muscle GlcCer ↑ hepatic glycogen levels, glycogen synthesis and insulin signal transduction attenuated inflammation in adipose tissue	(141)
CVDs	Egg yolk SM	C57BL/6 mice fed with HFD at 4 weeks old	0.3, 0.6, or 1.2% (wt/wt) egg yolk SM for 4 weeks	↓ Serum TMAO level – Serum choline and betaine levels	(30)
		apoE^–/–^ mice fed with HFD at 5 weeks old	1.2% (wt/wt) egg yolk SM for 16 weeks	↑ Body weight – Aortic lesions – Serum lipid levels (TC, TG, non-esterified fatty acid and SM)	
		apoE^–/–^ mice at 5 weeks old	1.2% (wt/wt) egg yolk SM for 19 weeks	↓ Aortic lesion area in the aortic arch ↓ Lesion composition – Serum lipid, TMAO, choline and betaine levels	
	Egg yolk SM	apoE^–/–^ mice fed with HFD at 6 weeks old	0.1% (wt/wt) egg yolk SM for 8 weeks	↓ Epididymal fat mass, alanine aminotransferase (ALT) activity ↓ Aortic root lipid accumulation ↑ Spleen weights – Serum TG, TC, HDL cholesterol, non-HDL cholesterol and non-esterified fatty acid levels	(128)
	Milk PLs	LDLr^–/–^ mice fed with AMF-rich diet at 6 weeks old	0.2%, 0.4% (wt/wt) milk SM for 14 weeks	↓ Serum TG, TC, HDL cholesterol, non-HDL cholesterol and non-esterified fatty acid levels – Serum HDL-C, TG, liver enzymes, insulin, glucose, TNF-α, IL-1β, adiponectin, and resistin ↓ Inflammatory markers in the serum, liver, adipose and aorta ↓ Atherosclerosis development in both the thoracic aorta and the aortic root ↑ Relative abundance of Bacteroidetes, Actinobacteria, and Bifidobacterium, and ↓ Firmicutes in cecal feces	(31)
	Milk PLs	Fifty-eight overweight postmenopausal women (ages 56–62)	Multi-center double-blind randomized trial with 4–6 weeks washout period 0, 3, or 5 g of milk PLs for 4 weeks	↓ Blood lipids and associated cardiometabolic risk markers (TC/HDL cholesterol, apoB/apoA1) ↑ Fecal loss of coprostanol – Major bacterial populations and fecal short-chain fatty acids ↓ Cholesterol absorption but ↑ cholesterol-ileal efflux	(32)
	Milk PLs	Fifty-eight overweight postmenopausal women (ages 56–62)	Multi-center double-blind randomized trial with 4–6 weeks washout period 0, 3, or 5 g of milk PLs for 4 weeks	↓ Serum TC, LDL cholesterol and apoB levels ↓ Serum atherogenic C24:1 Cer, C16:1 SM, and C18:1 SM species ↓ Chylomicron content in total SM and C24:1 Cer, but ↑ total Cer in feces	(148)

^*a*^Sphingolipids contained 68.2% ceramide-monosaccharide, 27.8% sphingomyelin, 2.9% cholesterol. ^*b*^Sphingolipids contained 64.8 % cerebroside and cerebroside sulfate, 29.0% sphingomyelin and 5.4% cholesterol and free fatty acids. ^*c*^↓ refers the significantly decreasing index, ↑ refers the significantly increasing index, and – refers no significant changes. SM, sphingomyelin; SLs, sphingolipids; ExHC, exogenous hypercholesterolemic; TG, triglyceride; CE, cholesterol esters; TC, total cholesterol; SD rat, Sprague-Dawley rat; So, sphingosine; NPC1L1, Niemann-Pick-Like Protein 1; SREBP-1/2, sterol regulatory element-binding transcription factor 1/2; HFD, high-fat diet; Adipor2, adiponectin receptor 2; PPARγ, peroxisome proliferator activated receptor alpha; Pdk4, pyruvate dehydrogenase kinase 4; Scd1, stearoyl CoA desaturase; LPS, lipopolysaccharide; MPL, milk polar lipids; LC-BS, lipid-concentrated butter serum; S1P, sphingosine-1-phosphate; PLs, phospholipids; SREBP-2, sterol regulatory element-binding protein-2; HMG-CoA, 3-hydroxy-3-methylglutaryl coenzyme A reductase; Cyp7a1, cholesterol 7α-hydroxylase; Elovl2, elongases of very long chain fatty acids 2; Fads2, fatty acyl desaturase 2; Cer, ceramide; GlcCer, glucosylceramide; TMAO, trimethylamine *N*-oxide; ALT, alanine aminotransferase; LDLr, LDL receptor; AMF, anhydrous milk fat.

**FIGURE 4:**
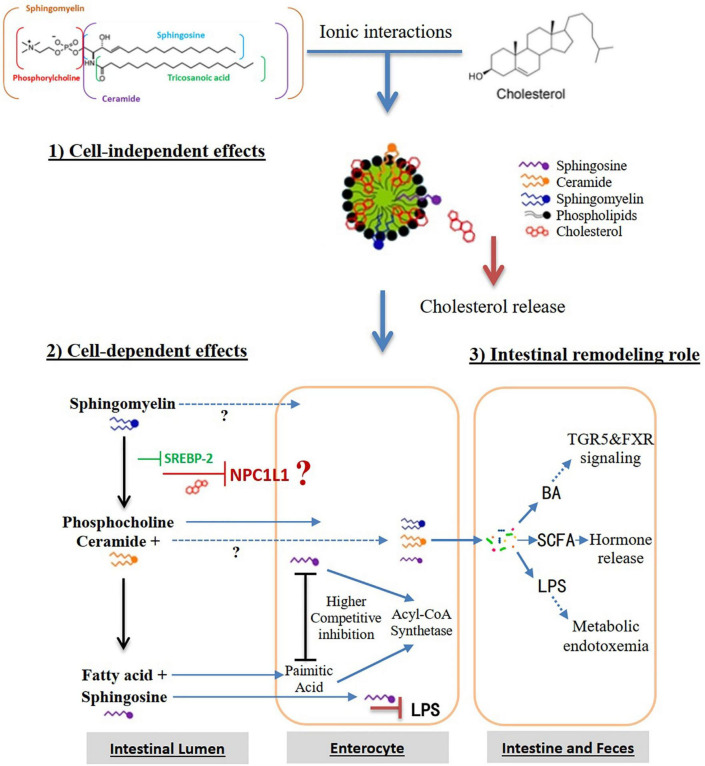
Hypothesis that dietary sphingomyelin inhibits the intestinal cholesterol absorption pathways. Alk-SMase, alkaline SMase; SREBP-2, sterol regulatory element-binding protein-2; NPC1L1, Niemann-Pick-Like Protein 1; N-CDase, neutral ceramidase; LPS, lipopolysaccharide; BA, bile acid; TGR5, Takeda G protein-coupled receptor 5; FXR, farnesoid X receptor; SCFA, short-chain fatty acids.

Compared with other phospholipids, sphingomyelin has a larger interface area and stronger hydrogen bond formation capability, which is essential for the interactions between sphingomyelin and other lipids in the cell membrane (54). The important intermolecular hydrogen bond is formed by the 2-NH or choline nitrogen of sphingomyelin and the hydroxyl group of cholesterol. The phosphate oxygen and the 3-OH among sphingomyelin molecules may also form intermolecular hydrogen bonds and a network, thereby interfering with the release of cholesterol from these lipid complexes (126). Nilsson et al. (127) found that about 25% of dietary sphingomyelin can reach to the colon, in the form of 10% undegraded sphingomyelin, 30–90% ceramides and 3–6% free sphingosine. Dietary sphingomyelin in the colon may have the potential for reducing the development of aortic root plaque (128), affecting the gut microbiota such as *bifidobacterial* (129), neutralizing the intestinal inflammation caused by lipopolysaccharide (LPS) and improving in the intestinal barrier function attenuated by Western diet (130). Based on these, we hypothesize that dietary sphingomyelin inhibits the intestinal cholesterol absorption pathways, as shown in Figure 4. (i) Cell-independent effects. The strong hydrogen bonding or interaction between sphingomyelin and cholesterol causes the molecules in the micelles to pack more tightly, thereby slowing down the release of cholesterol in the micelles and inhibiting the absorption and transport of cholesterol in the enterocytes. Sphingomyelin may reduce the solubility of cholesterol in micelles, thereby reducing the thermodynamic movement of free cholesterol that is available to be absorbed by enterocytes. (ii) Cell-dependent effects. Sphingomyelin inhibits cholesterol absorption by reducing the expression of NPC1L1 in the enterocytes. *In vitro* studies have shown that LCBs such as sphingosine compete with palmitic acid for absorption through an acyl-CoA synthetase-dependent mechanism (54). Acyl-CoA synthetases convert intracellular long-chain fatty acids to their acyl-CoA, which promotes their retention in the cells, and in turn, facilitates their transport. (iii) Intestinal remodeling role. Sphingomyelin may improve the distribution and abundance of gut microbiota, prevent LPS from being transported to the mesenteric lymphatic circulation, or modulate its BA and short-chain fatty acid (SCFA) pathways to inhibit cholesterol absorption and transport.

### Effects of dietary sphingomyelin on obesity

Obesity, the excess accumulation of body fat, has become a major public health issue worldwide. Overweight and obesity rates were respectively 34.3 and 16.4% between 2015 and 2019 in adults based on Chinese criteria (131). The sphingomyelin and its metabolites play a role in lipogenesis (132). Endogenous sphingomyelin is involved in adipose tissue functions and obesity-related pathology (24, 25). Exogenous dietary sphingomyelin may be beneficial for obesity prevention and treatment (28, 129, 133, 134). The sphingomyelin purified from chicken skin could significantly decrease hepatic lipid, plasma non-HDL cholesterol and insulin levels *via* improvement of adiponectin signaling such as adiponectin receptor 2 (*Adipor2*), peroxisome proliferator activated receptor alpha (*Ppara*), pyruvate dehydrogenase kinase 4 (*Pdk4*), and stearoyl CoA desaturase (*Scd1*) in Zucker rats fed a HFD (133). Dietary supplementation of sphingomyelin from egg yolk lowered the hepatic lipid content in mice fed a HFD, and intestinal cholesterol absorption content *via* inactivation of the LXR-SREBP-1c pathway, and increased fecal lipid and cholesterol output (28). Furthermore, dietary sphingomyelin from milk and egg yolk attenuated the HFD-induced hepatic steatosis *via* regulating the lipid absorption and metabolism, and lowered blood LPS *via* bifidogenic effects and alterations in distal gut microbiota (129, 134). Milk polar lipids containing 25% sphingomyelin may also affect gut physiology and the abundance of metabolically relevant bacteria in mice fed a HFD such as *Bifidobacterium* spp., *Akkermansia muciniphila* and *Lactobacillus reuteri*, which are associated with the fecal excretion of lipids and bile salts (135). In addition, the milk lipid concentrated-butter serum (LC-BS) and its main lipid fractions including ceramides and sphingomyelin can decrease TG and cholesterol levels in the liver and plasma of obese KK-A_*y*_ mice (136). These results indicate that dietary supplementation of sphingomyelin and ceramides may prevent fatty liver and hypercholesterolemia, and attenuate obesity.

### Effects of dietary sphingomyelin on diabetes

Insulin resistance in peripheral tissues is one characteristic for the occurrence and development of T2DM. Mitochondrial dysfunctions lead to the accumulation of lipid-derived metabolites such as ceramides, which may increase the risk of diabetes (137). Endogenous ceramides can cause T2DM and its chronic complications through a variety of ways. Therefore, either inhibition of ceramide *de novo* synthesis to enhance the mitochondrial functions in HFD-fed and *db/db* mice (26), or enhancement of the ceramide degradation to form S1P is a potential method to reduce ceramide level and attenuate lipotoxicity associated with it (138). For example, promoting ceramide degradation may reduce the synthesis of harmful lipids and promote synthesis of those beneficial ones such as S1P (138). The treatment with exogenous S1P significantly increases non-glucose stimulated insulin secretion in isolated mouse islets and HIT-T 15 cells *via* the activation of phospholipase C-Ca^2+^ system (139). The extracellular S1P can also attenuate the cytokine-induced apoptosis in pancreatic β cell through acting on its receptors, suggesting that S1P in the blood has potential protective effects *in vivo* (140). Milk sphingomyelin supplementation significantly increased fecal lipids, but lowered serum non-HDL cholesterol, hepatic TC and neutral lipids *via* regulating the mRNA levels of related genes for lipid metabolism in obese KK-A_*y*_ mice (29). Recently, treatments with exogenous glucosylceramides and ceramides from sea cucumber improve glucose tolerance and alleviate insulin resistance in high-fructose-diet-fed SD rats *via* upregulating the IRS/PI3K/Akt signaling pathway (141).

### Effects of dietary sphingomyelin on cardiovascular diseases

Atherosclerotic dyslipidemia, related to insulin resistance and visceral obesity, are important risk factors for CVDs (142). In the development of atherosclerosis, the endogenous sphingomyelin on the arterial wall can be degraded into ceramide by SMase, while the increase in ceramide content will promote the aggregation of lipoproteins (103). Studies have found that the content of ceramides in atherosclerotic LDL is 10–50 times of the normal level (143), and the endogenous sphingomyelin level in the plasma is correlated with atherosclerosis, which has been implicated as a risk factor for coronary artery disease (27). Therefore, the rate-limiting enzymes for the *de novo* synthesis of ceramides and sphingomyelin such as SPT andSMS1/2 may be targets for pharmacological intervention to prevent or treat atherosclerosis. The SMS2 and ApoE double knockout (KO) mice have lower sphingomyelin levels in lipoproteins, and contents of sphingomyelin, ceramides, free cholesterol, and cholesteryl ester contents in the brachiocephalic artery (144). Endogenous sphingomyelin synthesis promotes atherosclerosis. However, dietary sphingomyelin appears to prevent atherosclerosis by inhibiting cholesterol absorption (30, 118–120), modifying the plasma and hepatic cholesterol and TG metabolism (116, 117), and influencing lipoprotein formation and mucosal growth in the gut (14). Dietary sphingomyelin from egg yolk does not affect circulating sphingomyelin levels or increase atherosclerosis in apoE^–/–^ mice fed a HFD, but it is anti-atherogenic in apoE^–/–^ mice fed a normal chow diet, which may be related to sphingomyelin-mediated alterations in gut flora, a topic that needs further investigation (30). Dietary sphingomyelin from egg yolk reduced aortic neutral lipid accumulation in aortic root lipid accumulation of apoE^–/–^ mice fed a HFD, without affecting serum or hepatic lipids, as well as hepatic gene expression. Furthermore, there was a modest modulation of gut microbiota in apoE^–/–^ mice fed a HFD supplemented with sphingomyelin from egg yolk. It is worth noting that the mass ratio of sphingomyelin to cholesterol is 1:2 in the diet, which is similar to the ratio in egg yolk, indicating that egg yolk food matrix may have health benefits and/or counteract effects of dietary cholesterol (128).

Dietary sphingomyelin from milk also lowered atherogenic lipoprotein cholesterol, reduced inflammation and attenuated atherosclerosis development in LDLr^–/–^ mice fed a diet supplemented with anhydrous milk fat (31). Milk fat globule membrane (MFGM) comprises a core of TG surrounded by a structural membrane composed of phospholipids, cholesterol, proteins, and glycoproteins (145, 146). While compared with other animal-derived phospholipids containing lower than 5% sphingomyelin or plant-derived phospholipids without sphingomyelin, MFGM extracts have received widespread attention as a potential nutrient, especially their natural phospholipid composition (98, 147). A multi-center double-blind crossover study containing 58 overweight postmenopausal women (ages 56–62) shows that diets enriched with milk phospholipids can improve the cardiometabolic health by decreasing levels of blood lipids associated cardiometabolic risks and cholesterol absorption involving in specific interactions in the small intestine, and increasing cholesterol-ileal efflux and fecal loss of coprostanol without affecting the major bacterial phyla of gut microbiota and fecal SCFAs (32). In addition, diets enriched with milk phospholipids decreased the levels of blood ceramide (C24:1) and sphingomyelin (C16:1, C18:1) species to improve atherosclerosis, and increased total ceramides in feces (148).

## Application of dietary sphingomyelin in food

### Dairy products and infant formula

Urbanization and the general lack of time have shortened the nursing period despite great attempts to support breastfeeding for newborns. Only 35% of infants between the ages of birth and 6 months are breastfed globally (149). Substantial research has been conducted to study the potential application of MFGM in dairy products and infant formula (150–153). A recent review critically analyzed preclinical and clinical research data involving MFGM and its bioactive components in connection to the formation of gut microbiota, infection resistance, cognitive development, and infant metabolism (151). MFGM is highly organized and made up of a variety of elements, including polar lipids (phospholipids and glycosphingolipids), apolar lipids (cholesterol and cerebrosides) and specialized proteins (mostly glycoproteins) (154). Among them, sphingomyelin accounts for over 25% of milk phospholipids (145, 146). Sphingomyelin is an essential component of the milk phospholipid fraction owing to its high concentration of the MFGM and positive impacts on human health, including brain development in babies and protection of neonates from bacterial infections (155). Interestingly, long-chain SFAs (C16:0, C22:0, C23:0, and C24:0) with high melting points make up around 97% of all MFGM sphingomyelins. Rarely, milk sphingomyelins include C23:0 fatty acids in amounts of 17% or higher (154). This distinguishing feature of milk sphingomyelins points to a distinct, most likely a biological, function much beyond its straightforward structural function in the MFGM. Albi et al. (152) hypothesized that the sphingomyelin in human breast milk is important for regulating gene expressions and forming myelin sheets to assist the neural development. Data of clinical and mechanistic preclinical studies indicate that full-fat dairy diets reduce cardiometabolic risk through boosting gut health, decreasing inflammation, and regulating dyslipidemia. These cardiometabolic advantages at the gut and systemic levels are attributed, at least in part, to milk polar lipids produced from the phospholipid- and sphingolipid-rich MFGM, which is more abundant in full-fat dairy milk (153). Deficiency of choline, a metabolite of sphingomyelin, may contribute to the decreased gain of lean body mass and pulmonary and neurocognitive development of premature newborns despite receiving enough macronutrients and gaining body weight (156). At present, there are 3,939 patented inventions involving applications of sphingomyelin in infant formula milk worldwide.^1^ As brain development occurs between 0 and 3 years of age, these findings suggest a novel intervention strategy to include sphingomyelin into newborn formulae to create products equivalent to breast feeding. Consequently, more patented inventions relate to infant formulae containing large lipid globules and/or those coated with sphingomyelin such as United States Patent No. 11389403 are developed to support infant growth trajectories or physical development during the first year of life more similar to those infants fed the breast milk (157). The synthetic composition described in United States Patent No. 11297872 includes sphingomyelin, choline, CIP, and other glycosphingolipids, as well as metabolic precursors and metabolites in formula milk. This composition may be used to support or optimize *de novo* myelination, as well as brain structure, brain connectivity, intellectual potential, cognitive potential, learning potential, and cognitive enjoyment in a formula fed subject (158). The creation of a lipid formulation that resembles the human milk fat composition would be a big step for the baby formula business and would allow to produce high-tech formulas for newborns.

### Skin improvement

Any significant ultraviolet (UV) exposure sets off an inflammatory reaction in the epidermis, causing the skin to become dry and rough and compromising its barrier function (159). Photoaging, or prolonged exposure to UV radiation, affects both the dermal and epidermal layers of the skin, causing laxity, thickness, wrinkling, and changes in pigmentation (159, 160). Reactive oxygen species (ROS) are produced when skin is exposed to UV light. The oxidative damages due to the ROS production in keratinocytes and fibroblasts are reduced by protective and detoxifying non-enzymatic and enzymatic antioxidant molecules (161). Numerous dietary supplements have been shown to improve skin elasticity and health, including ascorbic acid, tocopherols, carotenoids, and polyphenols, which can be taken orally to prevent skin damages from UV radiation (159, 162, 163). The data of several animal research and clinical trials have shown the benefits of consumption of food-derived sphingomyelin and its’ metabolites on skin health, which include those derived from plants, animals, and marine species (164–171). In comparison to control mice, the supplementation of sphingomyelin (146 mg/kg body weight/day) considerably reduced covalently bound ω-hydroxy ceramides and significantly attenuated an increase in transepidermal water loss (TEWL) (165). Dietary sphingomyelin seems to further improve skin condition even under normal circumstances. Using hairless mice, Haruta-Ono et al. found that dietary milk sphingomyelin markedly decreased TEWL and stratum corneum hydration on epidermal conditions in the sphingomyelin-fed animals (166–168). Dietary soy sauce Lees ceramide, which was more efficient than maize glucosylceramide, also dramatically improved epidermal barrier function and reduced TEWL in normal hairless mice (169). Evidence from clinical trials suggests that regular, long-term orally supplementation with sphingomyelin may improve skin health (170, 171). Oral sphingomyelin supplementation dramatically improved volunteers’ perceptions of their skin’s condition, including the moisture of their face skin and the appearance of wrinkles around their eyes, as well as the hydration of the skin at the heel and the flexibility of the skin under the eye (170). Sphingomyelin, glucosylceramide, and lactosylceramide were the key components of the milk sphingolipid-enriched fraction cream, which helped the skin’s ability to retain water and maintain the barrier function (171). Experimental results suggest that the primary sphingoid bases in mammals, sphingosine and sphinganine from dietary sphingomyelin, may be partially reused in epidermal sphingolipids (168). Although the mechanism underlying skin condition enhancement is still not fully understood, previous studies have postulated some potential explanations. The fact that supplementary sphingomyelin reduced the covalently bound ω-hydroxy ceramides brought on by a single dose of UVB radiation is one reason for the mitigation of skin barrier deficiencies (165, 172). Dietary sphingolipids regulate the expressions of genes related to the development of the stratum corneum, which relates the production of the cornified envelope and tight junction proteins (173, 174). Another potential mechanism for the improvement of the skin barrier caused by dietary sphingomyelin is the reduction of the inflammatory response in the skin. According to Oba et al., in hairless mouse skin exposed to a single dose of UVB, the oral administration of milk sphingomyelin dramatically decreased the mRNA levels of genes linked to acute inflammation, such as thymic stromal lymphopoietin (TSLP), IL-1β, and IL-6 (165). The levels of thymus activation-regulated chemokine, and TSLP mRNA in hairless mice fed a magnesium-deficient diet were also markedly reduced by dietary milk sphingomyelin (172). Due to the functions of sphingomyelin and its metabolite ceramide to promote skin elasticity and health, there are more than 20,000 patents related to an effective ingredient of a skin beautifier and can be further blended with a food or feed to provide protection. Taking the United States Patent No. 20110065670 as an example, it relates to a meal or drink that improves the appearance of the skin as well as a skin beautifier that contains the useful element sphingomyelin and offers beauty benefits such skin moisturizing, skin beautification, wrinkle prevention, and skin roughness prevention (175).

### Delivery system

Vesicles, which include smaller vesicles, liposome, and exosomes with a diameter ranging from 30 to 100 nm, are biological entities encased by a membrane comprised of one or more lipid bilayers (176). Over the years, lipid vesicles have been regarded as effective medication delivery and carrier systems for bioactive compounds. Therefore, they are extensively employed in many different applications, including aesthetic, medicinal, therapeutic applications and growing food industry. Hydrophobic compounds can be solubilized inside of an aqueous vesicle since the vesicle is surrounded by a phospholipid bilayer (unilamellar vesicles or liposomes) or several concentric bilayers (multilamellar vesicles) (177). Lipid vesicles made of glycerophospholipids like soya lecithin or milk polar lipids, or model systems like dipalmitoyl-phosphatidylcholine, have been the primary focus of research for their potential use in the food industry (178, 179). Sphingomyelin molecules, the most abundant type of sphingolipid in biological membranes, perform structural functions and preferentially bind with cholesterol to create organized regions termed lipid rafts (126, 180). According to research by Morshed et al., lutein may be solubilized into the bilayers of egg-sphingomyelin vesicles, known as sphingosomes. This work opens up possibilities for the development of functional foods and drinks that are enriched with lutein and that have health benefits (177). The most thoroughly researched antimicrobial lipids are free fatty acids, monoglycerides, cholesteryl ester, sphingolipids, and others. Nano-sized lipid-based carriers may provide a tool of drug delivery for these antimicrobial agents, as they increase lipid solubility and dispersion in aqueous formulations. Additionally, nanocarriers may overcome drug resistance. Zhang et al. discussed several types of antimicrobial lipids in nanocarriers for the delivery of their activities. CAL02, a potential infection-controlling liposome composed of cholesterol and sphingomyelin, has been discussed as a novel anti-infection strategy, indicating that the antibacterial functions of antimicrobial lipids are worth to be investigated further (181). Sphingomyelin nanosystems are the promising form of carriers with potential for the combination of various types of medications (182–184). Bouzo et al. (182) encapsulated the anti-cancer drug etoposide in the sphingomyelin nanosystems, which mediated an antiproliferative response by interacting with colorectal cancer cells expressing the guanylyl cyclase receptor. Nagachinta et al. (183) designed sphingomyelin-based biocompatible nanosystems for intracellular delivery of microRNAs to colorectal cancer cells. Jatal et al. (184) revealed for the first time the possibility of combining sphingomyelin nanosystems with the 4N1Ks peptide for the development of novel senolytic cancer treatments. Therefore, sphingomyelin-based vesicles can be used to develop combination therapies. In addition, some inventions relate to the controlled delivery of different pharmaceutical agents (185–187). WIPO Patent No. WO/2001/041732 relates to compositions including sphingomyelins and methods for the intranasal delivery of active agents to the brain by means of neural pathways (188). WO/2022/047234 relates to devices and methods for the treatment of middle ear and/or inner ear disorders, and applicable to the treatment of other diseases in the human body. This kit includes sphingomyelins, stearyl, palmitoyl, tricosanyl sphingomyelins, ceramides, stearyl, palmitoyl ceramides, glycosphingolipids for delivering a drug formulation to treat diseases (189). The pharmaceutical agent protected by United States Patent No. 9186366 contains sphingomyelin and acts as a sialomucin secretion promoter, an antiallergic agent, an antioxidant, an infection defense agent, a hair growth agent, a therapeutic agent for demyelinating disease, an ant-pigmentation agent, an anti-inflammatory agent, or an agent that improves learning ability (190). As mentioned, sphingomyelin plays a role in cell structure, signaling, differentiation and functions. It is important to conduct more in *in vitro* and *in vivo* studies to assess the effectiveness of sphingomyelin vesicles in fortified foods.

### Oil organogels

In food applications, the demand for plastic fats from plants and animals such as margarine and shortening is on the rise. However, the contents of SFAs and *trans* fatty acids (TFAs) are relatively high in the plastic fats, which has been linked to a number of negative health effects, including altered lipoprotein profiles and a higher risk of MetS and CVDs (191, 192). This has promoted the research of oil organogels instead of traditional plastic fats in the food industry. The organogelation of liquid vegetable oils has become a hot spot in the research of food-specific oils with the purpose of reducing the content of SFAs and TFAs and increasing the intake of MUFA and polyunsaturated fatty acids (PUFA) in our diets (193). Organogelation of liquid vegetable oil is to fix or confine the liquid vegetable oil in a thermally reversible network to obtain a three-dimensional supramolecular network and liquid oil co-exist structure system, so that it has specific structural and functional properties which is called oil organogels (194). Oil organogels are structural oils constructed by applying less than 5% oleogelators to liquid oil including vegetable waxes, fatty acids, hydroxylated fatty acids, fatty alcohols, fatty acid–fatty alcohol mixtures (stearic acid and stearyl alcohol), fatty esters, monoglycerides, diacylglycerides, phospholipids, ceramides, sorbitan monostearate, phytosterols, sterols (γ-oryzanol and β-sitosterol), tocopherol mixtures, polyphenols mixtures, lecithin–sorbitan tristearate mixtures (193–200). According to Rogers et al. (194), the architecture of mixed ceramide networks can be easily tailored to closely resemble colloidal fat crystal networks, such as translucent ceramide-canola oil gels with 2% synthetically pure ceramide, or opaque enzymatically hydrolyzed egg yolk sphingomyelin-canola oil gels with 7% crude extracts. Rogers et al. found that 2% *N*-acetyl-D-erythro-sphingosine (C2) and other short-chain ceramides were able to form edible oils like canola oil into self-assembling organogels without the addition of SFAs and TFAs. The viabilities of colon, prostate, ovarian, and leukemia cell lines were reduced by C2 ceramide, but not C18 ceramide (196). Due to the obvious side chain characteristics, the melting of the organogelator can be adjusted according to the application to obtain functional properties. Sphingomyelin-derived ceramides obtained from dairy products, eggs, and soybeans are mostly long-chain ceramides. The high melting temperature of ceramides might be beneficial since they provide thermal stability to systems such as chocolate. However, their application as food-grade oleogelators is limited due to their extremely crystalline fibril structures and high melting points. Guo et al. added phosphatidylcholine to the mixed gel system of ceramides, and optimized the gel performance by changing the molar ratio of the components and the gel formation temperature (197–199). Generally, the effect of multi-component systems of oleogelators is often better than that of a single-component, and the gel performance can be optimized by changing the ratios of the components. In addition, some inventions relate to oleogelators by adding sphingomyelin and its metabolites in foods, beverages, nutraceuticals, pharmaceuticals, pet foods, or animal feeds (201–203). Ceramides have limited applications in food-grade oleogelators due to their crystalline fibril structures and high melting points, but a variety of sphingomyelin and derivatives can be used for the food-grade oleogelators. Future study may be conducted on the functions of monomers and multi-component compounds in oil organogels.

## Conclusion and perspectives

In this review, we summarized the content, structures, digestion, absorption, and metabolism of sphingolipids, and discussed the nutritional functioning of sphingomyelin in chronic metabolic diseases and the possible implications in food industry. Sphingolipids are a diverse category of chemically complicated molecules that are present in the brain, plasma, skin, and various foods including dairy, eggs, and soybeans. There is a need for a more comprehensive assessment of the varieties of sphingolipids in foods since certain diets may include adequate concentrations of atypical or typical sphingolipids that may have beneficial or detrimental impacts on human health. Many metabolites of sphingomyelin and derivatives are candidates for food-grade material, such as sphingoid bases, ceramides, C1P, whose structural formula are shown in Table 1. Sphingomyelin and ceramide are hardly absorbed in the small intestine. They can only be degraded into sphingosine to enter the circulation and be utilized by the body. Meanwhile, sphingoid bases, ceramides and sphingomyelin can also be interconverted, with ceramides being the central molecule. Ceramides can be desaturated to generate dihydroceramide, phosphorylated to form C1P and sphingomyelin, deacylated to generate sphingosine and then phosphorylated to produce S1P. Sphingomyelin is hydrolyzed by SMase to generate phosphorylcholine and ceramides, which in turn can be synthesized to C1P under the action of CerK. Phosphatidylcholine, sphingomyelin, and C1P are very similar in structure, with two long hydrophobic hydrocarbon chains and a polar head group. These characteristics of structure, absorption and metabolism can allow sphingomyelin and its metabolites to have wide applications in the food and pharmaceutical industry.

Clearly, dietary sphingomyelin benefits human health. Dietary sphingomyelin and its metabolites and derivatives have great potentials for clinical application in chronic diseases, cognitive development and intestinal health. Table 3 summarizes the ways by which dietary sphingomyelin may be effective in chronic metabolic diseases, including inhibiting cholesterol absorption, modifying the plasma hepatic cholesterol and TG metabolism, influencing lipoprotein formation and mucosal growth in the gut, improving glucose tolerance and alleviating of insulin resistance, and modulating gut microbiota community. In addition, sphingolipids are prevalent in the brain, where they generate cell-type-specific profiles that vary throughout development, aging, and pathological brain changes. Bioactive metabolic intermediates of gangliosides and sphingomyelin, especially ceramides and S1P, have emerged as crucial players in the preservation of brain health (204). The related papers mentioned in this review provide important references for the design of dietary sphingomyelin in the future.

When compared to other nutrients, dietary sphingolipids have drawn less attention and remain to be fully understood. Since no indication of sphingomyelin deficiency has been observed, it is not considered an essential nutrient. Many hypotheses have been proposed to explain the functional mechanism of sphingomyelin and its metabolites. However, many questions how dietary sphingomyelin and its metabolites influence a variety of intricate cell functions still remain unanswered. Future investigations are needed to determine the fate of the exogenously added sphingomyelin, and differentiate the roles of sphingomyelin itself and its metabolites. Especially, endogenous sphingomyelin is involved in pathological changes in obesity, diabetes and atherosclerosis; however, exogenous dietary sphingomyelin and its metabolites have been shown to maintain cholesterol homeostasis and lipid metabolism, as well as the prevention or treatment of chronic metabolic diseases. This seemingly paradoxical phenomenon needs to be further verified by rigorous clinical trials and its mechanism should be explored in more depth. Understanding of the underlying molecular mechanisms provide novel intervention strategies and target for the prevention and treatment of a variety of human illnesses. In general, the exciting but unexplained phenomenon shows that dietary sphingomyelin and its metabolites are candidates for food additives in food industry or natural medicines for metabolic related disease prevention.

## Author contributions

FY searched the publications, summarized the data in tables, drew figures, discussed the literature, and wrote the review. GC modified the review. Both authors read and approved the final version of this review.

## Footnotes

1
https://www.freepatentsonline.com/
